# 

**Published:** 1896-04

**Authors:** 


THE Twelfth International Medical Congress will be held in Moscow August 19th, continuing six days. The official announcement states that "papers may be read and discussed in the meetings in French, German, or Russian." The omission of English is an egregious and inexcusable blunder, which will result in a virtual exclusion of English-speaking physicians from the congress.

### EDITORIAL.

The office of the Business Manager of THE JOURNAL is Nos. 311 and 312 Fitten Building.

The editors are not responsible for views expressed by contributors.

Articles for publication should reach this office not later than the 15th inst.

Address all communications, and make all remittances payable to THE ATLANTA MEDICAL AND SURGICAL JOURNAL, Atlanta, Ga.

Reprints of articles will be furnished, when desired, at a nominal cost. Requests for the same should always be made *on the manuscript.*

### THE AMERICAN MEDICAL ASSOCIATION.

It is well known that this, the largest Association of American physicians and surgeons, will hold its annual meeting in Atlanta May 5th to 8th. We are informed that this meeting will be one of the most interesting and important in the history of the Association, and that there is now every indication of a large attendance. To those to whom this JOURNAL will come, we say: Lay aside your work for a few days and come up to witness the assembling of our leading doctors and teachers; mingle with them, listen to their discussions of medical and surgical questions, and you will go home pleased and better prepared for your labors. This will be an occasion which you cannot afford to miss. The journal of the Association says:

"A new era has dawned for American medicine, and the American Medical Association gives voice and expression to this more clearly than ever before.

"Local and State societies, and societies of specialists, all have a power and place in this movement, but the National society, of all others, must concentrate, mobilize, and direct this progress. The coming meeting will show a great advance in many ways, and higher ground will be occupied along many lines of science. The application in medicine of the new photography from the Roentgen rays will be presented with some new results. New discoveries of brain anatomy, and new facts along many practical lines of work, are already promised. The scientific outlook for the coming meeting is of unusual interest. National medicine has passed beyond any personal local influences and demands the best facts from every source. The village and country doctor is welcomed for any facts he may bring. Distinctions of place and position all become insignificant compared with truth and new facts and new evidence from any source.

"The many sectional meetings give every man a chance to compare his experience and opinions with others from all parts of the country.

"The Atlanta meeting will bring out a large number of active men in the new Southland who will be leaders in the years to come. All members from the West and North should embrace this opportunity to create a new scientific interest, and enlist a new class of men, who will bring new facts and new experiences to our work. It should be a duty and source of pride to every member of our Association to attend its annual meetings and prove to their home circle of friends that they are not merely medical hewers of wood and mechanical nurses, but scientific men interested in the great movements for the welfare of humanity.

"More than this, every member who attends the Association meetings secures a certain character and position, not only to himself personally, but to those he comes in contact with. This cannot be bought with money or power; it only comes from association and interest in medicine with others at these great national gatherings.

"Let every one show his interest in science, in humanity, and in the struggle against disease and death, and come to Atlanta, and he will go back stronger and better fitted for his work."

The officers of the association are: President, Dr. R. Beverly

Cole, California; Vice-Presidents, Dr. J. J. Chisolm, Maryland; Dr. J. C. LeGrand, Alabama; Dr. A. P. Clarke, Massachusetts; Dr. T. P. Saterwhite, Kentucky; Secretary, Dr. W. B. Atkinson, Pennsylvania; Treasurer, Dr. H. P. Newman, Illinois.

Following are the officers of sections for this meeting:

*Practice of Medicine*—Wm. E. Quine, Chicago, Ill., Chairman; DeLancey Rochester, Buffalo, N. Y., Secretary.

*Surgery and Anatomy*—C. A. Wheaton, St. Paul, Minn., Chairman; W. L. Estes, So. Bethlehem, Pa., Secretary.

*Obstetrics and Diseases of Women*—J. Tabor Johnson, Washington, D. C., Chairman; Reuben Peterson, Grand Rapids, Mich., Secretary.

*Ophthalmology*—Lucien Howe, Buffalo, N. Y., Chairman; Frank Alport, Minneapolis, Minn., Secretary.

*Laryngology and Otology*—G. V. Woolen, Indianapolis, Chairman; M. R. Ward, Pittsburg, Pa., Secretary.

*Diseases of Children*—A. C. Cotton, Chicago, Ill., Chairman; A. J. Work, Elkhart, Ind., Secretary.

*Materia Medica and Pharmacy*—F. E. Stewart, Detroit, Mich., Chairman; W. B. Hill, Milwaukee, Wis., Secretary.

*Physiology and Dietetics*—H. Bert Ellis, Los Angeles, Cal., Chairman; Henry Salzer, Baltimore, Md., Secretary.

*Neurology and Medical Jurisprudence*—T. D. Crothers, Hartford, Conn., Chairman; W. J. Herdman, Ann Arbor, Mich., Secretary.

*Dermatology and Syphilography*—L. D. Bulkley, New York City, Chairman; T. C. Gilchrist, Baltimore, Md., Secretary.

*State Medicine*—Chas. H. Shepard, Brooklyn, N. Y., Chairman; Elmer Lee, Chicago, Secretary.

*Dental and Oral Surgery*—R. R. Andrews, Cambridge, Mass., Chairman; Eugene S. Talbot, Chicago, Secretary.

The address of welcome will be delivered by Professor William

Osler, of Baltimore. The address on Surgery will be delivered by Professor Nicholas Senn, of Chicago, and Professor Geo. H. Rohe, of Baltimore, will deliver the address upon State Medicine.

The Chairman of the Committee of Arrangements is Dr. W. F. Westmoreland, Atlanta. The local Secretary is Dr. J. McF. Gaston, Jr.

The headquarters for visiting members and delegates will be the Hotel Aragon and Kimball House. The general sessions of the association will be held in the Grand Opera House. Halls for meetings of the sections have been secured.

*Let everybody come!*

---

### AMERICAN ACADEMY OF MEDICINE.

The twenty-first annual session of the American Academy of Medicine will be held in the dancing-hall of the Hotel Aragon, Atlanta, Ga., on Saturday, May 2d, and Monday, May 4th, 1896.

The proprietors of the Aragon have made special rates for those who attend the meeting. It is confidently expected that the concession for one and one-third fare for the round trip, granted to the American Medical Association, will be available in time for those who desire to attend the opening session. The secretary invites correspondence on any of these topics, and also from those who may desire a copy of the completed program, when it is issued; full information will be promptly given on any of these subjects.

The Academy will meet in executive session, with closed doors, on Saturday, May 2, at 10 A. M. The open session for the reading of papers will begin at about 11 A. M. There will be a recess for lunch from 1 to 2 P. M. The "reunion session" and annual dinner will be held on Saturday evening. An executive session will be held on Monday morning, after which the special discussion on "Methods of Medical Education" will be the order of the day.

The Association of American Medical Colleges and the Confederation of the State Boards of Medical Examiners and Licensers have accepted the invitation of the council, and will participate in this discussion.

Following is list of papers promised:

"Laboratories and Hospital Work," the President's address, Henry H. Hurd, M.D., Baltimore, Md.

"Colonies for Epileptics," Frederick Peterson, M.D., New York city.

"Insanity in the South," J. T. Searcy, M.D., Tuscaloosa, Ala.

"Tuberculosis in Public Institutions," J. W. Babcock, M.D., Columbia, S. C.

"Vivisection," George M. Gould, M.D., Philadelphia.

Subject not yet given, Woods Hutchinson, M.D., Iowa City, Ia.

"The Confusion of Pharmacy Relating to the Theory and Practice of Medicine," Elmer Lee, M.D., Chicago.

"A National Board to License for the Practice of Medicine," Henry Leffman, M.D., Philadelphia.

Report of the Committee to Abstract the Laws Regulating the Practice of Medicine and to Suggest a Model Law, Perry H. Millard, M.D., chairman, St. Paul, Minn.

"Homicide," Paul Bartholow, M.D., Philadelphia.

"The Sociologic and Scientific Attitude of the Medical Profession," W. J. K. Kline, M.D., Greensburg, Pa.

"A Study of Some of the Distinguishing Features of the Homo Medicus," Charles McIntyre, M.D., Easton, Pa.

The discussion on "Methods of Medical Teaching" will be opened by a series of ten-minute papers, and in the open discussion to follow each speaker will be limited to five minutes. The opening papers are as follows:

"The Preparatory Mental Discipline for the Medical Student," F. H. Gerrish, M.D., Portland, Me.

"The Lecture and its Uses," Charles B. Penrose, M.D., Philadelphia, Pa.

"Text-book Recitation and its Advantages," DeLancey Rochester, M.D., Buffalo.

"Laboratory Methods," V. C. Vaughan, M.D., Ann Arbor, Mich.

"Clinical Teaching for Graduates in Diseases of Children," J. Madison Taylor, M.D., Philadelphia.

"The Seminary Method," Bayard Holmes, M.D., Chicago.

"Examinations," E. L. Holmes, M.D., Chicago.

"Students' Medical Societies," Roswell Park, M.D., Buffalo.

"State Examination," J. McPherson Scott, M.D. (of the Maryland Board of Examiners), Hagerstown, Md.

"The Best Method to Teach Anatomy," John B. Roberts, M.D., Philadelphia.

"The best Method to Teach Physiology," Charles D. Smith, M.D., Portland, Me.

"The Best Method to Teach Practice," J. C. Wilson, M.D., Philadelphia.

"The Best Method to Teach Surgery," J. S. Wight, M.D., Brooklyn.

"The Best Method to Teach Obstetrics," J. C. Edgar, M.D., New York city.

"The Best Method to Teach State Medicine," George H. Rohe, M.D., Catonsville, Md.

Correspondence is pending about some additional papers; if any more are promised, the titles will appear in the completed program.

The Secretary of the Academy is Dr. Chas. McIntire, Easton, Pa.

### THE MEDICAL ASSOCIATION OF GEORGIA.

The forty-seventh annual session of the association will meet in Augusta April 15th, 16th, and 17th. It is hoped that there will be a large attendance of the State profession.

An interesting program of papers is being made up. Among those who have promised papers are: Dr. R. M. Harbin, Rome, "The After-Treatment of Tracheotomy Cases"; Dr. S. Latimer Phillips, Savannah, "Some Injuries of the Eyeball"; Dr. H. J. Williams, Macon, "Albuminuric Complications of Pregnancy"; Dr. W. F. Westmoreland, Atlanta, "Inguinal Hernia"; Dr. M. B. Hutchins, Atlanta, "Treatment of Skin Disfigurements by Electrolysis"; Dr. M. L. Boyd, Savannah, "Some Interesting Surgical and Gynecological Cases"; Dr. L. B. Grandy, Atlanta, "The Anesthetic"; Dr. A. G. Hobbs, Atlanta, "When do Adenoids and Polypi Cause Asthma and Hay Fever?"; Dr. E. H. Richardson, Atlanta, "The Medical Side of Appendicitis"; Dr. J. H. Shorter, Macon, "Asthenopia"; Dr. A. W. Sterling, Atlanta, "Glaucoma in Relation to General Practice"; Dr. C. H. Richardson, Montezuma, "The Best Treatment in Injuries of the Elbow-joint"; Dr. J. R. Shannon, Cabaniss, "Placenta Previa"; Dr. D. D. Quillian, Athens, "Suprapubic Operation for Fibroids"; and others. Dr. J. B. S. Holmes, Dr. F. W. McRae, and Dr. Bernard Wolff, of Atlanta, will read papers, titles not announced. This program will be very much extended by the time the association meets.

Be sure to go, and try to strengthen and build up your State Association.

---

THE ninth annual meeting of the National Association of Railway Surgeons will be held at St. Louis, April 30, and May 1 and 2, under the presidency of Dr. J. B. Murphy, of Chicago.

*We positively cannot send premium to any subscriber who does not pay a year in advance.*

NOTICE.—Our offer of a thermometer to *all* cash-in-advance subscribers is withdrawn. *New* subscribers, paying full year in advance, may still get the thermometer. Book offer still open to all who pay a year in advance. "Antisepsis and Antiseptics," "Stories of a Country Doctor."

